# The Edinburgh Postnatal Depression Scale (EPDS): translation and validation study of the Iranian version

**DOI:** 10.1186/1471-244X-7-11

**Published:** 2007-04-04

**Authors:** Ali Montazeri, Behnaz Torkan, Sepideh Omidvari

**Affiliations:** 1 Iranian Institute for Health Sciences Research, Tehran, Iran; 2 Faculty of Nursing and Midwifery, Khorasgan Azad University, Isfahan, Iran

## Abstract

**Background:**

The Edinburgh Postnatal Depression Scale (EPDS) is a widely used instrument to measure postnatal depression. This study aimed to translate and to test the reliability and validity of the EPDS in Iran.

**Methods:**

The English language version of the EPDS was translated into Persian (Iranian language) and was used in this study. The questionnaire was administered to a consecutive sample of 100 women with normal (n = 50) and caesarean section (n = 50) deliveries at two points in time: 6 to 8 weeks and 12 to 14 weeks after delivery. Statistical analysis was performed to test the reliability and validity of the EPDS.

**Results:**

Overall 22% of women at time 1 and 18% at time 2 reported experiencing postpartum depression. In general, the Iranian version of the EPDS was found to be acceptable to almost all women. Cronbach's alpha coefficient (to test reliability) was found to be 0.77 at time 1 and 0.86 at time 2. In addition, test-rest reliability was performed and the intraclass correlation coefficient was found to be 0.80. Validity as performed using known groups comparison showed satisfactory results. The questionnaire discriminated well between sub-groups of women differing in mode of delivery in the expected direction. The factor analysis indicated a three-factor structure that jointly accounted for 58% of the variance.

**Conclusion:**

This preliminary validation study of the Iranian version of the EPDS proved that it is an acceptable, reliable and valid measure of postnatal depression. It seems that the EPDS not only measures postpartum depression but also may be measuring something more.

## Background

Postpartum depression is very common among women and is a major public health problem [1]. However, there is a wide range of prevalence of postnatal depression among women from different countries. A recent review of 143 studies from 40 countries demonstrated that reported prevalence of postnatal depression ranged from almost 0% to almost 60% [2]. Thus, the recognition and assessment of this psychological disorder is important. The Edinburgh Postnatal Depression Scale (EPDS) is a brief and widely used instrument for measuring postnatal depression [3]. When it is used in non-postnatal women or men, the scale is referred to as the Edinburgh Depression Scale (EDS) [4]. It has been shown that the EPDS gives clinically meaningful results as a psychological screening tool. It is sensitive to change both during the course of pregnancy and after childbirth. A recent review of validation studies of the EPDS concluded that most studies reviewed showed high sensitivity for the EPDS, although uncertainty remained regarding the comparability between the sensitivity and specificity estimates of the different EPDS versions [5]. Recently a number of authors have proposed a shorter version of the EPDS. For example, using the Rasch analysis a revised 8-item version of the EPDS (EPDS-8) was suggested [6], or for non-postnatal women a short matrix-version (EDS-5) was recommended [7].

The EPDS is available in many languages such as French, Dutch, Swedish, Spanish, Chinese, Thai, Turkish and Arabic [8-14]. In general almost all studies reported that there were no problematic issues in translating the EPDS. For example, for the Dutch version it was reported that no important differences between the original and the translated version were seen [9]. Using the Spanish version of the EPDS for developing the Mexican version, the authors reported that since the Spanish version of the EPDS contained a number of words not currently used in Mexico, they have changed those words for more colloquial ones used for the general population in Mexico [15]. In addition, most validation studies of the EPDS reported satisfactory psychometric properties, although some authors indicated issues that need to be improved. For example, the validity values for Turkish version reported to be acceptable but not excellent and thus recommended that it needs to be improved for use in the Turkish population [13]. The aim of this study was to translate the EPDS to Persian (Iranian language), to validate and use the questionnaire in epidemiological and clinical studies.

## Methods

### Translation

The 'forward-backward' procedure was applied to translate the EPDS from English into Persian (Iranian language). Two health professionals translated the questionnaire into Persian and two professional translators backward translated these into English. Then, a provisional version of the Iranian questionnaire was developed and pilot tested and after review by a panel of experts (including the study coordinator, a translator, a midwife, a member of research team, a psychologist, and a psychiatrist); the final version of the questionnaire was provided [see Additional file 1]. In general there were no problematic issues concerning the translation process expect for item 10 that the panel agreed to use the Persian word 'suicide' instead of the less direct phrasing in the English 'The though of harming myself has occurred to me'. This was based on the judgment that word-by-word and direct translation of the phrase might not be understood by people in Iran. However, there were two concerns; one regarding with verb tenses and the other with translating response categories. The verbs in the original EPDS are 'present perfect' while in translation we used 'present' tenses giving almost similar meaning to the original instrument. With regard to response categories, the words 'yes' and 'no' were not used to avoid confusion. In Persian, for example, we usually do not use 'yes, most of the time' or 'no, never'.

### Sample and data collection

The final draft of the Iranian version of the EPDS was administered to a sample of 100 women with normal (n = 50) and caesarean section (n = 50) deliveries. The main intention to include these two groups was to examine whether the EPDS could discriminate between women with respect to their mode of delivery. We expected that women with caesarean section would show a higher score on the EPDS. Normal delivery was defined as unassisted vaginal delivery and the type of caesarean included both emergency and elective caesareans. The sample was recruited from 5 health care centers in Isfahan, Iran (a famous and historical city in the central part of Iran). Women were approached during their antenatal care. Those who agreed to take part in the study were listed for interview after childbirth. The sample size was based on an assumption that at least 10% of women in the normal delivery group and 18% of women in the caesarean group would suffer from postnatal depression. The figures were estimations and derived from a national study on mental health in the Iranian adult population [16]. As such, a study with a sample of 100 women (50 in each group) would have a power of 80% to detect a difference of 8% between two groups at the 5% significant level. A trained female nurse collected the data in face-to-face interviews at two points in time: 6 to 8 weeks and 12 to 14 weeks after delivery. The study received ethical approval from the Khorasgan Azad University and the Isfahan Health Authorities. All participants gave oral consent.

### Questionnaires

The EPDS contains 10 items and each item is rated on a four-point scale, giving maximum scores of 30. A score of 13 or more is considered to be a significant 'case' of postnatal depression, while scores of 10 to12 represent 'borderline' and 0 to 9 'not depressed' [3]. In addition to the EPDS, the validated Iranian version of the Short Form Health Survey (SF-36) also was administered [17]. This is a general measure of quality of life. Whilst not the focus of the present study; we used a data from the SF-36 for convergent analysis. Demographic data were collected using a short questionnaire at the women's first interview and included recording of age, educational level, employment status, and number of children as a proxy of childbirth experiences.

### Statistical analysis

Descriptive statistics including numbers, proportions, mean and standard deviations were used to present data. In addition the Chi-Square test was used for group differences.

To test reliability the internal consistency of the questionnaire was measured using Cronbach's alpha coefficient and alpha equal to or greater than 0.70 was considered satisfactory. Repeatability (test-retest reliability) of the EPDS was assessed using intraclass correlation coefficient (ICC). The ICC is an estimate of the fraction of the total measurement variability due to variation among individuals. We expected that the ICC for the EPDS items would exceed 0.7 [18].

Validity of the instrument was assessed using the known groups comparison and convergent analysis [19]. Known groups comparison analysis was examined to test how well the questionnaire discriminates between sub-groups of women who differed in mode of delivery. Convergent validity was carried out to demonstrate the extent to which the EPDS correlates with mental component summary score derived from the SF-36. It was expected that the EPDS would negatively correlate with this measure. Given the ordinal nature of the EPDS and the SF-36, the Spearman correlation was performed. The Spearman's correlation coefficient (*rho*) of 0.40 or above was considered satisfactory.

Finally, the factor structure of the questionnaire was extracted by performing principal component analysis using varimax factor solution. This is a rotation method that minimizes the number of variables that have high loadings on each factor. It simplifies the interpretation of the factors.

## Results

The characteristics of the women in the two groups are shown in Table 1. There were no significant differences between the two groups for variables studied. Almost all women (98%) found the Iranian version of the EPDS acceptable.

**Table 1:** The characteristics of women in two groups

	**Caesarean section (n = 50)**	**Normal delivery (n = 50)**	
	**No. (%)**	**No. (%)**	**P **(*df*, χ^2^)

Age groups*			0.83 (1, 0.04)
20–24	31 (61)	32 (64)	
25–29	17 (34)	16 (32)	
30–34	2 (4)	1 (2)	
35 ≥	0 (0)	1 (2)	
Mean (SD)	24.7 (3.17)	24.8 (3.68)	
Educational status*			0.85 (2, 0.32)
Illiterate	0 (0)	2 (4)	
Primary	13 (26)	11 (22)	
Secondary	28 (56)	30 (60)	
Higher	9 (18)	7 (14)	
**Employment**			0.56 (1, 0.33)
Housewife	42 (84)	44 (88)	
Employed	8 (16)	6 (12)	
**Number of children**			0.31 (1, 1.02)
One	31 (62)	26 (52)	
Two	19 (38)	24 (48)	

* To carry out valid Chi-Square test, the cells were merged.

The internal consistency of the EPDS as measured by the Cronbach's alpha coefficient was found to be *0.77 *at time 1 (6–8 weeks after delivery) and *0.86 *at time 2 (12–14 weeks after delivery) indicating a satisfactory reliability. Overall 22 % of women at time 1 and 18% at time 2 reported symptoms of postnatal depression. The results are shown in Table 2. In addition, in test-retest analysis, the ICC was found to be satisfactory (*ICC = 0.80*).

**Table 2:** The EPDS score for all women at two points in time and the Cronbach's alpha coefficients as indicates the scale reliability

	**Time 1 (6–8 week after delivery, n = 100)**	**Time 2 (12–14 weeks after delivery, n = 100)**
	**No. (%)**	**No. (%)**

**EPDS score**		
0–9 (not depressed)	63 (63)	67 (67)
10–12 (borderline)	15 (15)	15 (15)
13–30 (case ness)	22 (22)	18 (18)
Mean (SD)	8.5 (5.1)	7.6 (5.7)
*Cronbach's alpha*	*0.77*	*0.86*

Validity of the EPDS was examined using the known groups comparison and convergent analysis. The EPDS discriminated well between sub-groups of women as defined by their mode of delivery indicating that as expected depression score was higher in women with caesarean section delivery. Although not significant, women who had experienced a caesarean were more likely to be depressed compared with women who had normal births. The results are shown in Table 3. Convergent validity was assessed using the correlation between the EPDS score and mental health component summary score of the Iranian version of the Short Form Health Survey (SF-36) and as expected a significant negative correlation emerged (at time 1: Spearman's *rho *= -0.41, P < 0.001; at time 2: Spearman's *rho *= -0.57, P < 0.001).

**Table 3:** The EPDS scores for women in two groups in two points in time

	**Time 1 (6–8 weeks after delivery)**		**Time 2 (12–14 weeks after delivery)**	
	**Caesarean section (n= 50)**	**Normal delivery (n = 50)**	**Caesarean section (n = 50)**	**Normal delivery (n = 50)**

	**No. (%)**	**No. (%)**	**No. (%)**	**No. (%)**

**EPDS scores**				
0–9 (not depressed)	27 (54)	36 (72)	31 (62)	36 (72)
10–12 (borderline)	10 (20)	5 (10)	9 (18)	6 (12)
13–30 (case ness)	13 (26)	9 (18)	10 (20)	8 (16)
Mean (SD)	9.3 (5.1)	7.8 (4.9)	8.1 (5.3)	7.2 (6.1)
*P (df, χ*^2^*)*	*0.15 (2, 3.68)*		*0.55 (2, 1.2)*	

The principal component analysis with varimax rotation was performed and a three-factor structure jointly accounted for 58% of the variance. The results are shown in Table 4. Apart from item 8 (I have felt sad or miserable that loaded with both factor 1 and factor 2), other items loaded in three distinct factors producing factors of 'euthymic mood', 'anxiety' and 'depression'.

**Table 4:** Factor structure of the EPDS using principal component analysis with varimax rotation solution

	**Factor 1**	**Factor 2**	**Factor 3**
EPDS items*			
**1. **I have been able to laugh and see the funny side of things	0.07	0.05	**0.77**
**2. **I have looked forward with enjoyment to things	0.06	0.07	**0.76**
**3. **I have blamed myself	**0.64**	0.04	0.06
**4. **I have been anxious or worried for no good reason	**0.79**	0.09	0.13
**5. **I have felt scared or panicky for no very good reason	**0.78**	0.19	- 0.01
**6. **Thing have been getting on top of me	0.35	**0.48**	0.34
**7. **I have been so unhappy that I have had difficulty sleeping	0.37	**0.61**	- 0.24
**8. **I have felt sad or miserable	**0.52**	**0.52**	0.33
**9. **I have been so unhappy that I have been crying	0.28	**0.76**	0.08
**10. **The thought of harming myself has occurred to me	- 0.19	**0.71**	0.12
**% of variance**	33.7	13.1	11.2

* All items loading on a factor are shown. Item loadings of ≥ 0.4 were considered satisfactory.

## Discussion

The EPDS is a well-known instrument for measuring postnatal depression. However, it is not a tool for indicating specific diagnosis. This study reports data from a validation study of the EPDS in Iran. In general, the findings showed promising results and were comparable with most research findings throughout the world [8-15]. The Iranian version of the EPDS proved to be acceptable to women and similar to most studies, its reliability as measured by the internal consistency and test-retest analysis was found to be satisfactory. Furthermore, there is evidence that the English version of the questionnaire can be understood and completed in similar ways by English-speaking women of non-English-speaking backgrounds and when carefully translated is also likely to be a valid measure in cross-cultural research on depression following childbirth [20].

The known groups comparison indicated that the Iranian version of the EPDS is a valid instrument for measuring depression in women since the instrument was able to discriminate between women who differed in mode of delivery. However, despite our earlier expectations the study findings showed no significant differences in postpartum depression between women who had caesarean and vaginal deliveries. This is in fact in line with recent evidence of no significant differences in postpartum depression between women who had vaginal or caesarean deliveries [21].

The results of this study showed that the EPDS includes three factors expressing euthymic mood, anxiety, and depression. Studies have shown that the EPDS contains two factors: depressive feelings, and anxiety [8,9,22]. The later study [22] reported that because item 10 (the thought of harming myself) clearly was different from the other items, a principal component analysis without this item still yielded an anxiety and depressive symptoms subscales. Similarly a study performed both exploratory and confirmatory factor analyses also revealed that the EPDS to be comprised of distinct and correlated anxiety and depression subscales [23]. A three-factor solution also was identified for the EPDS in a study reporting anxiety (items 3, 4, 5, 6, and 7), depression (items 8, 9, and 10), and anhedonia (items 1, and 2) as components of the questionnaire accounting for 63% of the variance [24]. Also a recent study indicated that the EPDS contains three factors: one principal factor (depression) and two supplementary factors (loss of enjoyment, and despair/self-harm) [20]. However, one may argue that this is evidence to suggest that the instrument is a general measure of psychological distress rather than a one-dimensional measure of depression. As suggested in fact the EPDS captures elements of anxiety and depression that it is generally accepted that these symptoms frequently occur together, and assessment of both should be identified as a clear clinical need in the antenatal and postnatal periods [25,26]. Thus, this could be regarded as a strength for the EPDS, a questionnaire that is short and easy to use in both research settings and clinical practice.

Using the recommended cut points by the EPDS developers, the findings from this preliminary validation study showed a higher level of postpartum depression in Iranian women as compared to their counterparts in other countries. We suspect if we had been be able to compare outcomes on the EPDS with a gold standard such as psychiatric diagnosis this could even indicate more depression in Iranian women after childbirth. Thus, further investigation might be necessary and indeed using an objective criteria or a gold standard test is needed to answer this question. Unfortunately, the present study was limited in this respect. Without such analysis the EPDS may result in under-estimation or over estimation of psychiatric morbidity and therefore its utility for screening purposes might be limited before establishing a validated cut-off score [5,27]. With respect to choosing cut-off scores for women from non-English speaking backgrounds it has been recommended that if no studies have been conducted, there is need to describe the rationale for whatever score is used [27].

The EPDS showed a negative significant correlation with the mental component summary score of the SF-36 as expected. This means that those who were more depressed showed lower levels of mental health. Thus, this could be regarded as additional evidence to suggest that the EPDS is a valid questionnaire. In some validation studies concurrent validity analysis was applied using the correlation between The EPDS and the Beck Depression Inventory (BDI), or the General Health Questionnaire (GHQ), or the State-Trial Anxiety Inventory (SATI) [11,22,28]. However apart from the GHQ-12, since at the time of the present study there were no validated Iranian versions of these questionnaires we used the mental component summary score of the validated Iranian version of the SF-36. Interestingly a study on cross-cultural experiences of maternal depression among Vietnamese, Turkish and Filipino women in Victoria, Australia also used and compared the EPDS with the SF-36 findings [29].

## Conclusion

In summary, the findings from this preliminary validation study indicate that the Iranian version of the EPDS is a reliable and valid measure of depression in puerperal women. In addition, it seems that the EPDS not only measures postpartum depression but also it may be measuring something more.

## Competing interests

The author(s) declare that they have no competing interests.

## Authors' contributions

BT designed the study, and collected the data. AM analyzed the data, and wrote the paper. SO contributed to the interpretation of the findings. All authors read and approved the final manuscript.

## Pre-publication history

The pre-publication history for this paper can be accessed here:



## Supplementary Material

Additional File 1 Iranian (Persian) version of the EPDS. The file contains the Iranian version of the Edinburgh Postnatal Depression Scale. Click here for file
