# Supplementary material for: The Edinburgh Postnatal Depression Scale (EPDS): translation and validation study of the Iranian version
Source: BMC Psychiatry. 2007 Apr 4;7:11. doi: 10.1186/1471-244X-7-11 (PMC1854900; doi:10.1186/1471-244X-7-11)
Supplement: Additional File 1 — Iranian (Persian) version of the EPDS. The file contains the Iranian version of the Edinburgh Postnatal Depression Scale. [file 1471-244X-7-11-S1.doc]

**The Edinburgh Postnatal Depression Scale (EPDS)**

# گونه ايراني

|  |  | |
| --- | --- | --- |
| از آنجا كه شما حامله هستيد يا تازه بچه دار شده ايد ، مي خواهيم بدانيم شما چه احساسي داريد. لطفاً جمله اي را كه به احساس شما نه تنها در حال حاضر بلكه در 7 روز گذشته نزديكتر است علامت بزنيد . | | |
| 6. مسائل و مشكلات بيش از حد توان من هستند.  🞎 بيشتر اوقات نمي توانم با مشكلات بسازم.  🞎گاهي اوقات نمي توانم با مشكلات بسازم.  🞎 بيشتر اوقات به خوبي با مشكلات مي سازم.  🞎 مثل هميشه به خوبي با مشكلات مي سازم. | | **1**. من مي توانم بخندم و جنبه هاي خنده دار چيزها را ببينم.  🞎 مثل هميشه  🞎 نه مثل هميشه  🞎 قطعاً نه مثل هميشه  🞎 اصلاً |
| **7** .آنقدرغمگين هستم كه به سختي مي توانم بخوابم.  🞎 بيشتر اوقات  🞎 گاهي اوقات  🞎 كمي از اوقات  🞎 اصلاً | | **2** . من هميشه منتظر وقايع شادي آور هستم.  🞎 مثل هميشه  🞎 كمي كمتر از هميشه  🞎 خيلي كمتر از هميشه  🞎 اصلاً |
| **8** . من احساس غم و درماندگي مي كنم.  🞎 بيشتر اوقات  🞎 گاهي اوقات  🞎 كمي از اوقات  🞎 اصلاً | | **3**. وقتي چيز ها بر وفق مرادم نيست بي دليل خودم را سرزنش مي كنم.  🞎 بيشتر اوقات  🞎 گاهي اوقات  🞎 كمي از اوقات  🞎 اصلاً |
| **9**. از شدت ناراحتي گريه مي كنم.  🞎 بيشتر اوقات  🞎 گاهي اوقات  🞎 بندرت  🞎 هرگز | | **4**. بدون دليل موجه مضطرب و نگران هستم.  🞎 اصلاً  🞎 بندرت  🞎 گاهي اوقات  🞎 بيشتر اوقات |
| **10**. فكر خود كشي به ذهنم مي آيد.  🞎 بيشتر اوقات  🞎 گاهي اوقات  🞎 بندرت  🞎 هرگز | | **5**. بدون دليل خوبي احساس ترس و وحشت دارم.  🞎 بيشتر اوقات  🞎 گاهي اوقات  🞎 كمي از اوقات  🞎 اصلاً |
| **©** استفاه از اين پرسشنامه در پژوهش هاي علمي منوط به كسب اجازه كتبي از دكتر علي منتظري ( پژوهشكده علوم  بهداشتي جهاد دانشگاهي) يا بهناز تركان (دانشكده پرستاري و مامايي دانشگاه آزاد اسلامي خوراسگان) است*.*  *پديد آورندگان :*  Cox JL, Holden JM, Sagovsky R. Detection of postnatal depression. Development of the 10-item Edinburgh Postnatal Depression Scale. Br J Psychiatry 1987; 150: 782-786. | | |
